# The Gaps Between Current Management of Intracerebral Hemorrhage and Evidence-Based Practice Guidelines in Beijing, China

**DOI:** 10.3389/fneur.2018.01091

**Published:** 2018-12-11

**Authors:** Di Li, Haixin Sun, Xiaojuan Ru, Dongling Sun, Xiuhua Guo, Bin Jiang, Yanxia Luo, Lixin Tao, Jie Fu, Wenzhi Wang

**Affiliations:** ^1^Beijing Neurosurgical Institute, Capital Medical University, Beijing, China; ^2^Beijing Municipal Key Laboratory of Clinical Epidemiology, Capital Medical University, Beijing, China; ^3^School of Public Health, Capital Medical University, Beijing, China

**Keywords:** intracerebral hemorrhage, management strategies, international practice guidelines, gap, questionnaire

## Abstract

**Background:** The leading cause of death in China is stroke, a condition that also contributes heavily to the disease burden. Nontraumatic intracerebral hemorrhage (ICH) is the second most common cause of stroke. Compared to Western countries, in China the proportion of ICH is significantly higher. Standardized treatment based on evidence-based medicine can help reduce ICH's burden. In the present study we aimed to explore the agreement between the management strategies during ICH's acute phase and Class I recommendations in current international practice guidelines in Beijing (China), and to elucidate the reasons underlying any inconsistencies found.

**Method:** We retrospectively collected in-hospital data from 1,355 ICH patients from 15 hospitals in Beijing between January and December 2012. Furthermore, a total of 75 standardized questionnaires focusing on ICH's clinical management were distributed to 15 cooperative hospitals. Each hospital randomly selected five doctors responsible for treating ICH patients to complete the questionnaires.

**Results:** Numerous approaches were in line with Class I recommendations, as follows: upon admission, all patients underwent radiographic examination, about 93% of the survivors received health education and 84.5% of those diagnosed with hypertension were prescribed antihypertensive treatment at discharge, in-hospital antiepileptic drugs were administered to 91.8% of the patients presenting with seizures, and continuous monitoring was performed for 88% of the patients with hyperglycemia on admission. However, several aspects were inconsistent with the guidelines, as follows: only 14.2% of the patients were initially managed in the neurological intensive care unit and 22.3% of the bedridden patients received preventive treatment for deep vein thrombosis (DVT) within 48 h after onset. The questionnaire results showed that imaging examination, blood glucose monitoring, and secondary prevention of ICH were useful to more clinicians. However, the opposite occurred for the neurological intensive care unit requirement. Regarding the guidelines' recognition, no significant differences among the 3 education subgroups were observed (*p* > 0.05).

**Conclusions:** Doctors have recognized most of ICH's evidence-based practice guidelines. However, there are still large gaps between the management of ICH and the evidence-based practice guidelines in Beijing (China). Retraining doctors is required, including focusing on preventing DVT providing a value from the National Institutes of Health Stroke Scale and Glasgow Coma Scalescores at the time of admission.

## Introduction

In most Western countries, after coronary heart disease and cancer, stroke represents the third most common cause of death (1). However, it has been the leading cause of death in recent years in China (2). Nontraumatic intracerebral hemorrhage (ICH) is the second most common cause of stroke, carrying a higher risk of mortality and disability (3, 4). An earlier study has shown through a meta-analysis of 36 trials that the median case fatality at 1 month was 40%, and the long-term functional independence rate was only 12–39%. An improvement over time was not observed for either parameter (5). In China, 17–55% of strokes are caused by ICH (6, 7), which is a higher proportion compared to that in Western countries (8, 9). Standardized treatment based on evidence-based medicine can help reduce ICH's disease burden. To date, there are only two studies available on ICH's current management in China (10, 11). However, such studies have not focused on the consistency between clinical practice in ICH's management and the recommendations in the current evidence-based international practice guidelines.

The clinical practice guide is defined as the best guidance and is obtained from a systematic synthesis of the evidence generated and an evaluation of the pros and cons of the various options for intervention. In such a guide, a level I recommendation implies evidence that supports and/or agrees that an operation or treatment is useful and effective. In the present study, we systematically examined the current management and functional outcome of ICH patients in Beijing. Additionally, we focused on assessing the consistency between clinical management of the acute phase of ICHand the guidelines for the management of spontaneous ICH from the American Heart Association/American Stroke Association in 2010. Furthermore, we analyzed the underlying reasons of any inconsistencies found.

## Methods

### Study Design

We retrospectively collected in-hospital data of ICH patients from 15 hospitals in Beijing between January and December 2012. The hospitals were selected as follows. Firstly, the hospitals were classified as grade I (community hospitals with only the most basic facilities and very limited inpatient capacity), grade II (hospitals with at least 100 inpatient beds providing acute medical care and preventative care services to populations of at least 100,000), and grade III hospitals (major tertiary referral centers in the provincial capitals and major cities) in China. Since grade I hospitals are unable to treat ICH patients, they were not included in the study. Secondly, the Public Health Information Center has registered all medical institutions in Beijing, and there are 121 hospitals that can treat patients with ICH, including 71 grade II and 50 grade III hospitals. Thirdly, we divided 121 hospitals into groups according to geographical location, that is, their districts and counties. Lastly, one hospital was randomly selected in each group and the hospital agreed to participate in the investigation. As a result, we included eight grade II and seven grade III hospitals. Patients were traced from the discharge lists with an ICH diagnosis. We retrospectively investigated the clinical data and treatment information.

Additionally, a questionnaire for doctors was developed and focused on the clinical management of ICH, referring to guidelines for the management of spontaneous ICH and the hospital facilities, including hospital imaging equipment, related treatment drug stocks and ward conditions, etc. A total of 75 standardized questionnaires were distributed to 15 cooperative hospitals. Each hospital randomly selected five doctors responsible for treating ICH patients to complete the questionnaires. All surveys on ICH patients and doctors were completed by March 2014.

### Patient Eligibility

Following the World Health Organization criteria (12), diagnosis of ICH was performed by using computed tomography (CT) or magnetic resonance (MR) imaging. ICH patients were eligible for the study if they met the following criteria: (1) diagnosis of spontaneous ICH; (2) age ≥18 years; and (3) presentation within 7 days from onset of symptoms. We excluded from the study any ICH case secondary to trauma.

### Clinical Data

The demographic and clinical variables were are follows: sex, age, body mass index, alcohol, and tobacco use as well as history of hypertension, diabetes mellitus, hyperlipidemia, stroke, coronary heart disease, or medications (antihypertensive, antiplatelet, and anticoagulation agents). Stroke severity upon admission was evaluated using the Glasgow Coma Scale (GCS) and the National Institutes of Health Stroke Scale (NIHSS).

The enrolled hospitals utilized the following diagnostic and management approaches: routine laboratory tests, neuroimaging, intravenous/oral medications, supportive care, and surgery. Additionally, we recorded the length of hospital stay, the modified Rankin Scale score, health guidance at discharge, death, and the discharge destination. A poor clinical outcome was defined as modified Rankin Scale >2 at discharge.

Finally, we recorded whether there were in-hospital complications (e.g., pneumonia, deep venous thrombosis [DVT], recurrent stroke, urinary tract infection, sepsis, pulmonary embolism [PE], coronary event, seizure, and fall with injury) or any other clinically significant events responsible for prolonging the hospital stay.

### Radiological Data

We recorded the presence or absence of intraventricular extension, the localization of hematomas and the hematomas' volume. The latter was recorded based on the ABC/2 method, in which A is the greatest diameter on the largest hemorrhage slice; B is the diameter perpendicular to A; and C is the approximate number of axial slices with hemorrhage multiplied by the slice thickness (13). The hematomas' localization was subclassified as follows: basal ganglia, lobar, thalamus (supratentorial), cerebellum, brainstem (infratentorial), or intraventricular.

### Doctor Questionnaire Data

There were three parts to the doctor's questionnaire. The first part involved the participants' demographic variables. The second part involved information of the hospitals they visited. The third part involved investigating factors associated with ICH management. The item pool for the questionnaire was generated from Class I recommendations of guidelines for the management of spontaneous ICH from the American Heart Association/American Stroke Association in 2010. The third part of the questionnaire required a specific rating on a four-point scale. Specifically, this was based on the clinicians' assessment of the need for the items in the questionnaire: (1) no reason to be done; (2) could be done or not; (3) should be done; and (4) must be done. Additionally, a final open question was included. With the latter, clinicians were asked to explain the reason behind the choice of an item that should be done or must be done. We calculated the scores by summing the ratings of all participants for each item.

### Quality Control and Data Collection

All sub-centers received the centrally designed standardized paper-based case report form. Physicians responsible for the sub-centers were trained centrally by the task group. Once trained, the physicians were then responsible for training the first-level monitors at the sub-centers, following a uniform protocol. During the course of the study, 20% of the case report forms were randomly selected by the independent clinical research organization and were compared to the original case information in order to verify data authenticity. A team of on-site staff supervised the administration of the questionnaires in each sub-center. The participants independently completed the questionnaire after having received instructions by a researcher. Additionally, the researcher supervised the completed questionnaire to ensure that all questions had been answered. Completeness and logical consistency were evaluated in all the data submitted to the task group. Finally, the task group utilized double entry for the data.

### Statistical Methods

Statistical analyses were performed using the statistical package version 19.0 (SPSS, Inc., Chicago, IL, United States). Continuous variables were summarized as median (interquartile range) or mean (SD). Categorical variables were presented as percentages. To compare the scale scores, we used the following tests: one-way analysis of variance; the Mann–Whitney *U*-test; and the Kruskal–Wallis *H*-test. All tests were two-tailed, and statistical significance was given to *p* < 0.05.

## Results

Following a review of the discharge lists, 1,442 patients were observed to have a diagnosis of spontaneous ICH. Of these, 87 patients were excluded from this study. The reasons for the exclusion were as follows: (1) two patients were < 18 years of age; (2) 36 patients were hospitalized 7 days after the onset; and (3) 49 patients were missing data. The final statistical analysis included the remaining 1,355 patients. Table 1 describes the demographics and clinical characteristics of ICH patients. The median age was 60.7 years, and 64.1% of the patients were men. The proportion of all ICH patients with a history of hypertension was 68.9%. The frequency of bleeding sites for all ICH patients was as follows: 46.4% basal ganglia; 20.1%, lobar; 17.8%, thalamus, 6.3%, brainstem; 6%, cerebellum; and 3.4% intraventricular hemorrhage.

**Table 1:** Characteristics of ICH patients*.

	**All *N* = 1355(%)**
Age, years, mean (SD)	60.7(13.6)
Male	869 (64.1)
Living alone	44 (3.2)
**EDUCATION**	
Elementary or below	429 (31.7)
Middle school	321 (23.7)
High school or above	605 (44.6)
**DISEASE HISTORY**	
Hypertension	933 (68.9)
Diabetes mellitus	186 (13.7)
Hyperlipidaemia	52 (3.8)
Stroke	362 (26.7)
Atrial fibrillation	145 (10.7)
Coronary artery disease	113 (8.3)
Current smoker	323 (23.8)
Current heavy drinker^§^	63 (4.6)
Overweight^||^	869 (64.1)
**MEDICATION HISTORY**	
Antihypertensive therapy	470 (34.7)
Antiplatelet therapy	90 (6.6)
Warfarin	18 (1.3)
Lipid-lowering therapy	21 (1.5)
Hypoglycaemic therapy	117 (8.6)
**CLINICAL FEATURES**	
Time from symptom onset to hospital presentation < 5 h	744 (54.9)
**Blood pressure, mmHg, median (IQR)**
Systolic blood pressure	172 (167–176)
Diastolic blood pressure	95 (85–105)
**Laboratory, median (IQR)**
Hemoglobin, g/DL	140 (134–150)
White cell count, 10^9^/L	8.1 (6.1–9.4)
Platelets, 10^9^/L	209 (177–302)
INR	0.9 (0.7–1.3)
Serum glucose, mmol/L	7.9(5.7–11.2)
**Imaging characteristics**	
**Haematoma location**
Basal ganglia	629 (46.4)
Lobar	272 (20.1)
Thalamus	241 (17.8)
Brain stem	86 (6.3)
Cerebellum	81 (6)
IVH	46 (3.4)
**Haematoma volume (mL)**^‡^	
**Supratentorial**
< 30	868 (66.3)
30–60	231 (17.6)
>60	43 (3.3)
**Infratentorial**
< 10	123 (9.4)
10–20	34 (2.6)
>20	10 (0.8)
Intraventricular extension	560 (42.9)

IQR, interquartile range; INR, international normalized ratio; IVH, intraventricular hemorrhage; *Values are reported as mean ± SD, median (IQR), or number (percentage) of subjects.

§ *≥4 drinks on any single day prior to stroke onset (a standard drink was defined as 12 fl oz of regular beer, 5 fl oz of table wine, or a 1.5–fl oz shot of 80-proof spirits)*.

|| *Defined as body mass index (BMI) ≥24*.

‡ *46 patients with IVH were excluded*.

Table 2 shows that 689 patients were transported to the hospital by ambulance, and the assessment proportions of the NIHSS and GCS scores upon admission were only 8.9 and 15.3%, respectively. A total of 194 patients (14.3%) received cerebral vascular imaging (e.g., CTA/MRA/CTV/MRV) and the following secondary causes were identified: tumor, arteriovenous malformations, aneurysms, cavernous hemangioma, and moyamoya disease in 57 cases. A total of 1,129 patients (83.3%) underwent imaging reexaminations at the hospital. Additionally, of a total of 28 patients with ICH caused by coagulopathy, 19 (67.9%) received one or more reversal treatments with platelets, vitamin K, fresh-frozen plasma (FFP), or prothromb in complex concentrates (PCCs). Among 991 patients who were bedridden within 48 h from onset, 221 (22.3%) received preventive DVT treatment. Specifically, seven patients were simultaneously managed by intermittent pneumatic compression (IPC) and elastic stockings (ES), while 186 and 28 patients were managed with IPC and ES, respectively.

**Table 2:** In-hospital management and outcomes of ICH patients.

	**Actual treatments number of cases**	**Should be taken number of cases by guideline/All Number**	**%**
Transported to hospital by ambulance	689	1,355	50.8
Assessment NIHSS score on admission	121	1,355	8.9
Assessment GCS score on admission	208	1,355	15.3
**NEUROIMAGING ASSESSMENT**			
CTA/MRA/CTV/MRV	194	1,355	14.3
Review of neuroimaging (CT/MRI)	1,129	1,355	83.3
**HEMOSTASIS/ANTIPLATELET TREATMENT**			
Coagulopathy^§^	28	-	-
Reversal therapy by one or more of platelet, vitamin K, FFP, and PCCs	19	28	67.9
**DVT PROPHYLAXIS**			
Bedridden within 48 h after onset	991	-	-
One or more of IPC and ES	221	991	22.3
**General monitoring**		
Treated at the Neuro-ICU	193	1,355	14.2
Treated at the Stroke unit	66	-	-
Treated at the Neurology ward	458	-	-
Treated at the Neurosurgery ward	473	-	-
**MANAGEMENT OF GLUCOSE**			
Random blood glucose ≥11.1 mmol/L on admission	343	
Glucose monitoring within 24 h of onset	302 (22.3)	343	88.0
Received hypoglycemic therapy mainly with insulin	267 (19.7)	343	77.8
**MANAGEMENT OF SEIZURES**			
Clinical seizures or electroencephalography seizures	61	-	-
Patients presented with seizures received antiepileptic treatment	56	61	91.8
Patients presented without seizures received prophylactic antiepileptic treatment	95	-	-
**NEUROSURGICAL INTERVENTION FOR CEREBELLUM HEMATOMA**			
Indications for surgery	22	-	-
Received surgery	11	22	50.0
**OUTCOME AT DISCHARGE (AS MEASURED BY THE MODIFIED RANKIN SCALE)**			
0–2	429	1,355	31.7
3–5	594	1,355	43.8
6	332	1,355	24.5
**PREVENTION OF RECURRENT ICH FOR SURVIVORS AFTER DISCHARGE**			
Health education	950	1,023	92.9
Antihypertensive treatment	595	704	84.5

*NIHSS, National Institutes of Health Stroke Scale; GCS, Glasgow Coma Score; CTA, CT angiography; CTV, CT venography; MRA, magnetic resonance angiography; MRV, magnetic resonance venography; CT, computerized tomography; MRI, magnetic resonance imaging; FFP, fresh frozen plasma; PCC, prothrombin complex concentrates; DVT, deep vein thrombosis; IPC, intermittent pneumatic compression; ES, elastic stockings; Neuro-ICU, neurological intensive care unit*.

§ Defined as platelet ≤ 70^*^10^9^/L or international normalized ration ≥2.0.

Only 193 patients (14.2%) were initially treated in the neurological intensive care unit (Neuro-ICU). The remaining patients were admitted to the neurology and neurosurgical wards. Of the 343 patients exhibiting hyperglycemia upon admission, 88% were continuously monitored and 77.8% received hypoglycemic therapy, mostly as insulin. Sixty-one patients (4.5%) presented with clinical seizures or electroencephalography (EEG) seizures. Of these, 56 patients (91.8%) received antiepileptic drugs during hospitalization. Additionally, 95 cases (7.0%) received a prophylactic antiepileptic treatment. EEG and bedside EEG were used in 22 (1.6%) and 12 (0.9%) patients, respectively. A total of 22 patients had a cerebellar hematoma with indications for surgery. Of these, 11 patients (50%) received surgical intervention; six cases underwent combined hematoma evacuation with decompressive craniectomy/shunt insertion, four cases underwent shunt insertion alone, and one underwent decompressive craniectomy alone. The in-hospital mortality rate was 24.5% (332/1355). Among the 1,023 survivors, 594 patients (Modified Rankin Scale 3–5) were disabled/dependent at discharge. Approximately 93.0% of the survivors received health education and 84.5% of those diagnosed with hypertension were prescribed antihypertensive treatment at discharge.

Here we summarize the infrastructures of 15 hospitals. All hospitals were usually equipped with CT, MR imaging (MRI), and EEG, and had platelets, FFP, and IPC available. Only nine hospitals (60.0%) had the emergent green channel for ICH. Hospitals rarely had PCCs and recombinant factor VIIa availability (Table 3). As shown in Table 4, the 75 doctor questionnaire respondents mostly had a master's degree, and the proportion of those with both a master's and doctoral degree was 76.0% of all respondents. Table 5 shows that, regarding the guidelines, most clinicians agreed with the utility of examining patients with imaging and utilizing antihypertensive therapy as a preventive for recurrent ICH. However, most clinicians did not consider Neuro-ICU management as a requirement in ICH's acute phase.

**Table 3:** Characteristics of cooperative hospital related to ICH management^*^.

**Variable**	**All *N* = 15(%)**
Emergent green channel for ICH	9 (60)
Neuro-ICU	10 (66.7)
**EQUIPMENT/PREPARATION**	
CT	15 (100)
MRI	15 (100)
Platelets	15 (100)
FFP	14 (93.3)
PCCs	3 (20)
rFVIIa	1 (6.7)
ES	12 (80)
IPC	14 (93.3)
EEG	15 (100)
Bedside EEG	8 (53.3)

*ICH, intracerebral hemorrhage; Neuro-ICU, neurological intensive care unit; CT, computerized tomography; MRI, magnetic resonance imaging; FFP, fresh frozen plasma; PCCs, prothrombin complex concentrates; rFVIIa, recombinant activated human coagulation factor VII; IPC, intermittent pneumatic compression; ES, elastic stockings; EEG, electroencephalogram*.

* *Values are reported as number (percentage) of subjects*.

**Table 4:** Characteristics of doctor questionnaire respondents^*^.

	**All *N* = 75**
**SEX**	
Male	40 (53.3)
**AGE (YEARS)**	
20–40	59 (78.7)
41–60	16 (21.3)
**HIGHEST LEVEL OF EDUCATION**	
Graduate	18 (24)
Master	43 (57.3)
Doctor	14 (18.7)
**DEPARTMENTS**	
Neurology	45 (60)
Neurosurgery	30 (40)
**PROFESSIONAL TITLE**	
Junior	26 (34.7)
Intermediate	27 (36)
Senior	22 (29.3)

* *Values are reported as number (percentage) of subjects*.

**Table 5:** Results of the 10-item questionnaire to assess the perceived as important of the AHA guideline recommendations^*^.

**Abbreviated item-label**^§^	**All** ***N*** **=** **75 (%)**			
	**A**	**B**	**C**	**D**
Rapid neuroimaging assessment	0	0	4 (5.3)	71 (94.7)
Rapid hemostasis/antiplatelet treatment for deficiency	1 (1.3)	1 (1.3)	29 (38.7)	44 (58.7)
Rapid correction of coagulopathy	0	0	16 (21.3)	59 (78.7)
Deep vein thrombosis prophylaxis	0	3 (4.0)	29 (38.7)	43 (57.3)
Treatment in Neuro-ICU	1 (1.3)	28 (37.3)	11 (14.7)	35 (46.7)
Glucose monitoring and normoglycemia	0	1 (1.3)	14 (18.7)	60 (80)
Antiepileptic therapy for clinical seizures	3 (4.0)	3 (4.0)	12 (16)	57 (76)
Antiepileptic therapy for electrographic seizures	1 (1.3)	6 (8)	19 (25.4)	49 (65.3)
Neurosurgery for severe cerebellum hematoma	0	0	21 (28)	54 (72)
Antihypertensive treatment for prevention of recurrent ICH	0	0	1 (1.3)	74 (98.7)

*A, represents no reason to be done; B, represents could be done or not; C, represents should be done; D, represents must be done*.

* *Values are reported as number (percentage) of subjects*.

§ represents the question: should the following items be conducted for ICH patients?

No significant difference was observed by analysis of variance among the three education groups (*p* > 0.05; Table 6). Additionally, the Mann–Whitney *U*-test found no significant differences among the two department groups (*p* > 0.05). Finally, we did not observe significant differences among the three title groups by the Kruskal–Wallis *H*-test (*p* > 0.05).

**Table 6:** Subgroup analyses comparing total scores of participants for each item.

	**Scores**	**Test Value**	***P***
**HIGHEST LEVEL OF EDUCATION, MEAN ±*SD***			
Graduate (*n* = 18)	32.6 ± 2.9	
Master (*n* = 43)	33.1 ± 2.3	0.49	0.61
Doctor (*n* = 14)	33.4 ± 2.7	
**DEPARTMENTS, MEDIAN (IQR)**			
Neurology (*n* = 45)	35 (34–39)	−1.690	0.091
Neurosurgery (*n* = 30)	39 (36–40)	
**PROFESSIONAL TITLE, MEDIAN (IQR)**			
Junior (*n* = 26)	33 (31–34)	
Intermediate (*n* = 27)	34 (32–36)	3.36	0.187
Senior (*n* = 22)	34 (31.5–35)	

*IQR, interquartile range*.

## Discussion

Previously reported national multicenter studies focused exclusively on a few sub-centers from Beijing (10) or a disproportionate number of grade II and III hospitals (11). Such evaluations could not truly reflect the status of ICH management in Beijing. Furthermore, in these rigorously designed prospective studies, sub-centers conducted research based on uniform case report forms and flowcharts. This resulted in biases and overestimation of the patterns of care. On the contrary, in the present study, only 9 and 15% patients were evaluated with NIHSS and GCS scores, respectively, on admission by doctors. These observations were far from the lowest proportions reported in other studies (10, 11). Similarly to community residents, EMS was called by 689 (51%) patients following the onset of symptoms (14). Nevertheless, we believe that our results are valid and may be more representative of actual realistic scenarios. Additionally, in this study, the elaborate questionnaire was of aid in revealing the underlying reasons that led to the inconsistencies between current management in the acute phase of ICH and evidence-based guidelines.

The Class I recommendations in the guidelines include the following: pre-hospital transfer, emergency diagnosis and assessment, medical treatment, inpatient management and prevention of secondary brain injury, procedures of clot removal, prevention of recurrent ICH, etc. (15). As for emergency diagnosis and assessment, brain imaging is the gold standard for diagnosing ICH because clinical presentation alone is unable to differentiate hemorrhagic from ischemic stroke (16–20). In the present study, we implemented a rapid neuroimaging assessment. As a consequence, none of patients were excluded due to lack of CT/MRI. This approach was in line with the current practice guidelines. Additionally, 14% of the patients underwent cerebral vascular imaging. The results obtained from the doctor's questionnaires demonstrated that all hospitals were generally equipped with both CT and MRI. Clinicians agreed on the utility of these diagnostic modalities for ICH's diagnosis. The frequent use of CT/MR angiogram supported the high reported interest in rapidly identifying ICH's etiology. Unfortunately, we observed that the proportion of CTA/MRA examinations was very low. Therefore, this finding indicates that strengthening the doctors' education on the use of CTA/MRA is needed.

The guidelines recommended rapid correction of severe coagulopathy. In the present study, approximately 68% of the patients with thrombocytopenia or coagulopathy upon admission received reversal treatment. The questionnaires' results demonstrated that over 95% of clinicians agreed that correction should or must be done. However, the clinicians found that limited care and “do not resuscitate” orders were a common reality. As a consequence, the latter measures represented a limitation to the standard scheme's application. Additionally, while previous studies have shown that PCC administration was optimal for rapid coagulopathy reversal (21), in the present study, FFP and vitamin K were used with a higher frequency. Such a discrepancy could be due to two reasons. First, the clinicians were unfamiliar with the use of PCCs and management of PCCs' complications. Second, only a few hospitals in Beijing had PCCs available, as opposed to FFP and vitamin K, which were generally more available.

Previous studies found that the combination of ES and IPC was associated with a reduced risk of asymptomatic DVT compared to ES alone. Furthermore, ES alone was insufficient to prevent DVT (22, 23). In the present study, only 22% of the bedridden patients received preventive DVT treatment within 48 h of onset. Such a finding was extremely inconsistent with the guidelines. Additionally, none of the cases in the ES combined with the IPC group were complicated with DVT. However, in this study eight cases of DVT were observed in the ES or IPC alone groups. Such an observation suggests that ES or IPC alone did not represent an appropriate preventive measure. The questionnaires' results demonstrated that over 95% of the clinicians agreed with DVT prevention, but exclusively for bedridden patients within 48 h from onset. However, a considerable number of clinicians preferred intermittent alternative means to ES or IPC for DVT prevention (e.g., extremity massage). This may represent one of the reasons underlying the poor outcomes.

In a previous study, Diringer et al. observed an increase of the in-hospital mortality rate in ICH patients who were not managed in the Neuro-ICU, finding a lower mortality rate in those patients who were under the full-time care of an intensive care unit (24). In the present study, only 14% of the patients were initially managed in the Neuro-ICU. Such a percentage was extremely inconsistent with the guidelines. The questionnaire's responses showed that only 60% of the clinicians agreed that Neuro-ICU management should or must be done. The remaining clinicians believed that Neuro-ICU management for ICH patients depended on their severity and clinical course (e.g., in the case of a hematoma enlargement). Additionally, it is of note that only a handful of tertiary hospitals in Beijing have an independent Neuro-ICU. Such ward conditions may offer insufficient space for the accommodation of all ICH patients. Finally, the high cost involved in Neuro-ICU management may also represent a potential barrier.

It has been demonstrated that high blood glucose upon admission predicts an increased risk of mortality and poor outcome in ICH patients with and without diabetes (25–27). Of note, studies with tight glucose control on ICH patients have shown contradictory results (28–32). To date, the optimal management of hyperglycemia in ICH and the target glucose remain unclarified. In the present study, the vast majority of patients with hyperglycemia on admission received continuous monitoring and hypoglycemic therapy. This approach was highly consistent with the guidelines. This might be due to the fact that the implementation of measures was relatively easy. As shown in the questionnaires, 99% of the clinicians unanimously agreed that hyperglycemic management should or must be done.

It has been shown that the incidence of seizures post-ICH varies widely, from < 10% to >20% (33–36). Numerous seizures were detected exclusively by EEG monitoring. Earlier studies have demonstrated that exclusively clinical seizures or EEG seizures in patients with a change in mental status should be treated with antiepileptic drugs (37, 38). In the present study, most of the patients that presented with seizures received antiepileptic drugs during their hospital stay. Additionally, prophylactic antiepileptic treatment was administered to patients receiving surgical intervention. The questionnaire's results showed that >90% of the clinicians agreed that antiepileptic drugs should or must be administered in the event of seizures. Of note, prophylactic antiepileptic therapy has been shown to be harmful for ICH patients (37, 38). However, its universal application in surgical patients suggests that on this matter, neurosurgeons in Beijing might prefer habitual practices rather than rely on the guidelines. Our findings show that epilepsy is diagnosed in < 5% of patients, perhaps because of the low use of EEG and bedside EEG.

Current guidelines recommend that patients with cerebellar hemorrhage, who are deteriorating neurologically or who have brainstem compression and/or hydrocephalus from ventricular obstruction, should undergo surgical removal of the hemorrhage as soon as possible. However, none of these recommendations are based on prospective clinical trials with clearly defined selection criteria (39–42). In the present study, surgical intervention was performed on 50% of the patients with indications for surgery. This might due to the higher ratio of hematoma without IVE and mild compression of the fourth ventricle in this patient group. The questionnaire's results showed that 100% of the clinicians agreed that surgery should or must be indicated in these cases. However, the optimal timing for surgery remained controversial because perihematomal edema fluctuated over time.

Following ICH's acute phase, the large randomized PROGRESS trial found that blood pressure reduction was beneficial in the prevention of future vascular events (43). The effect was particularly strong for ICH's secondary prevention; an average systolic blood pressure reduction of 12 mmHg decreased the risk of recurrent ICH by up to 76% (44). In this study, most of the survivors received health education. This was especially true for patients with hypertension who continued to receive antihypertensive treatment post-discharge. The questionnaire's results showed that 100% of the clinicians agreed that antihypertensive treatment should or must be prescribed. However, in reality, care was generally limited, especially for patients with a poor clinical outcome.

Our study has some limitations that deserve comments. First, our study may be affected by selection bias because this hospital-based study emphasized moderate-to-severe strokes that required admission, while rapidly fatal or very mild strokes may not have been directly admitted into the hospital. Second, as the Chinese capital, Beijing has relatively better medical resources than most other parts of the country, so we assume that there is a huge gap in other areas. Third, this study was a cross-sectional study that lacked follow-up information. Therefore, the association between management patterns and long-term functional outcomes post-ICH was not evaluated. Besides, the patient's treatment information occurred in 2012 and the doctors were questioned in early 2014. There was indeed a lag of more than 1 year between the two parts of the surveys. Fourth, our study focused on exploring if clinical practices in Beijing conformed to Class I recommendations in the guidelines for ICH management. Additionally, there were largely low-level recommendations that we did not assess, such as the following: temperature management, intracranial pressure monitoring and treatment, surgical intervention for supratentorial hematoma, etc. Last, in this study, the assessment proportion of the NIHSS and GCS scores on admission was so low that the existing data do not represent the stroke's severity.

In summary, while most of the evidence-based practice guidelines of ICH have been recognized by doctors, there are still large gaps between current ICH managements and evidence-based practice guidelines in Beijing, China. Therefore, strengthening doctors' education and aiding the development of management strategies to improve ICH care are needed. With its massive population undergoing rapid aging and other demographic transitions, China faces an increasingly heavy burden of stroke across a variety of healthcare settings. The health status of Beijing is likely a microcosm of that of China. Therefore, having a better understanding of current management strategies can help to develop and improve the healthcare process for patients with ICH in China.

## Ethics Approval and Consent to Participate

This study was approved by the Institutional Review Board of Tiantan Hospital Affiliated to Capital Medical University of China (IRB No: KY2014-044-03). The acquisition of informed consent was exempted because patients' identification was not uncovered, and there was no intervention such as drug therapy in this retrospective analysis.

## Author Contributions

DL, HS, and WW conceived and designed the experiments. DL, HS, XR, and DS performed the experiments. DL, HS, YL, LT, and JF collected and analyzed the data. DL and HS wrote the manuscript. XG, BJ, and WW revised the manuscript.

### Conflict of Interest Statement

The authors declare that the research was conducted in the absence of any commercial or financial relationships that could be construed as a potential conflict of interest.

## Abbreviations

BMI: body mass index
CT: computed tomography
CTA: CT angiography
CTV: CT venography
DVT: deep venous thrombosis
EEG: electroencephalogram
ES: elastic stockings
FFP: fresh frozen plasma
GCS: Glasgow Coma Scale
ICH: intracerebral hemorrhage
INR: international normalized ratio
IPC: intermittent pneumatic compression
IQR: interquartile range
IVH: intraventricular hemorrhage
MR: magnetic resonance
MRA: magnetic resonance angiography
MRI: magnetic resonance imaging
MRV: magnetic resonance venography
Neuro-ICU: neurological intensive care unit
NIHSS: National Institutes of Health Stroke Scale
PCCs: prothrombin complex concentrates
PE: pulmonary embolism
rFVIIa: recombinant factor VIIa.
